# A matched case-control study on the interaction of anxiety, depression, and circadian CLOCK genes (CLOCK, PER2, RORA) in sleep disorders among mental workers

**DOI:** 10.3389/fpubh.2025.1579151

**Published:** 2025-08-27

**Authors:** Xiaoting Yi, Xiaofan Ma, Lingyun Shi, Xue Li

**Affiliations:** ^1^Linghu People’s Hospital, Huzhou, China; ^2^Department of Public Health, Xinjiang Medical University, Ürümqi, China; ^3^First Affiliated Hospital of Xinjiang Medical University, Ürümqi, China

**Keywords:** mental workers, anxiety, depression, sleep disorders, circadian clock genes

## Abstract

**Objective:**

The current status of the occurrence of anxiety, depression, and sleep disorders in mental workers was investigated. The effects of anxiety, depression, and CLOCK, PER2, and RORA gene polymorphisms and their interactions on sleep disorders were further analyzed, to provide scientific references for the reduction of the risk of the occurrence of sleep disorders in mental workers.

**Methods:**

Anxiety, depression, and sleep disorders in the study population were measured by applying the Self-Assessment Scale for Anxiety (SAS), the Self-Assessment Scale for Depression (SDS), and the Pittsburgh Sleep Quality Index (PSQI). The CLOCK, PER2, and RORA genes of 748 mental workers (374 of whom were randomly selected from the sleep disorder group and 374 of whom were randomly selected from the normal sleep group) were genotyped by imLDR™ genotyping technology, and the relationship between CLOCK, PER2, and RORA gene polymorphisms and their interactions with sleep disorders were analyzed.

**Results:**

The detection rate of sleep disorders among mental workers was 27.88%. There were significant differences in the rates of sleep disorders among mental workers of different genders, ages, marital status, shifts, education, title, occupation, and monthly income (*p* < 0.05). There was a difference in the prevalence of sleep disorders between groups with different levels of anxiety and depression (*p* < 0.001). Anxiety and depression scores were positively related to PSQI scores (*r*_s_ = 0.626, *r*_s_ = 0.661, *p* < 0.001) and their scores in all dimensions. The rs10462028 and rs11932595 of the CLOCK gene, the rs934945 of the PER2 gene, and the distribution of genotypes and allele frequencies of each genotype, as well as allele frequency, were significantly different in the sleep-disordered group and the normal-sleep group (both *p* < 0.001). The difference in distribution was also significant (all *p* < 0.05). The interaction of rs934945, anxiety, and depression (OR = 10.461, 95% CI: 3.695–29.621) increased the risk of sleep disorders in mental workers (*p* < 0.05).

**Conclusion:**

Mental workers experience significant sleep disorders, so effective measures should be taken to reduce anxiety and depression. The interaction of rs934945, anxiety, and depression was associated with a higher prevalence of sleep disorders in mental workers.

## 1 Background

The rapid development of the social economy, the continuous advancement of globalization, and the rapid changes in emerging technologies have all posed significant challenges to contemporary workers (1). As the main force of productivity, the physical and mental health of workers has a very important impact on the development of the economy and social stability, and poor physical and mental health among workers will lead to a low work capacity, reduced sleep quality, frequent production accidents, and can even cause huge social and economic losses (2). At present, an increasing number of scholars are interested in the negative health effects caused by psychosocial factors, and previous studies have shown that the incidence of occupational stress and psychological disorders in laborers has not only gradually increased but also seriously jeopardized the quality of their sleep and their physical and mental health (3–5). Therefore, it is particularly important to pay attention to the impact of psychosocial factors on the physical and mental health of workers and to improve the current situation.

Anxiety and depression, as the most common disorders resulting from mental health conditions, occur widely in all types of populations and have high lifetime prevalence rates (6). A U. S. Census Bureau survey indicated that the prevalence of anxiety and depression among U. S. adults in 2020 was three times higher than in 2019 (7). Globally, depression has been identified as a leading cause of ill health and disability (8), and the World Health Organization (WHO) has estimated that depression will be the world’s highest-burden disease in the next decade (9).

Sleep, as a biological process, takes up one-third of the human life cycle, ensures the continuity of human health, and is considered one of the most basic physiological needs of humans (10). Normal healthy sleep is characterized by a long duration, good quality, appropriate timing, regularity, and the absence of sleep disorders (11). Sleep disorders are chronic conditions characterized by the inability to fall asleep normally or to maintain sleep continuity, resulting in poor subjective sleep satisfaction (12). The high prevalence of sleep disorders is a major problem in modern society, and epidemiologic surveys have shown that approximately one-third of the general adult population in Europe reports having a sleep disorder (13, 14). The results of several studies have confirmed that sleep disorders, such as poor sleep quality, insufficient sleep time, sleep disruption, difficulty falling asleep, and insomnia, lead to an increased risk of chronic diseases (e.g., diabetes mellitus, hypertension, and obesity), as well as psychiatric disorders (e.g., anxiety disorders, depression, etc.) (15–17). Sleep disorders can also seriously affect the work efficiency of laborers, leading to a decrease in productivity and an increased occurrence of accidents in the production process (18). Therefore, it is urgently necessary to pay more attention to the occurrence of sleep disorders in occupational groups.

In recent years, due to the rapid development of molecular biology, a large number of scholars have delved into sleep-related studies examining general demographic and psychosocial factors as well as genetic factors. The circadian biological clock network (central and peripheral oscillators) controls circadian rhythms and coordinates the expression of a series of genes that enable the organism to anticipate and adapt to environmental changes (19). Biological rhythms are composed of two parts: the exogenous part, regulated by environmental factors (20), and the endogenous part, related to genetic factors (21). Currently, CLOCK, PER, CRY, and BMAL1 are the major circadian clock genes found in humans, and there are other clock genes involved in circadian rhythm regulation, such as RORA, RORB, and casein kinase-1 (CK1). Circadian clock genes alter 24-h rhythms by affecting endogenous circadian rhythms and desynchronization between sleep–wake cycles, which can increase the risk of developing sleep disorders (22). Polymorphisms (SNPs) in clock genes have been associated with the development of anxiety disorders, depression, and sleep disorders, and it has been found that circadian clock genes have a wide range of physiological effects on cognition, mood, and sleep behavior and that mutations in them can lead to mood disorders, imbalances in the sleep–wake cycle, and changes in the organism’s metabolic levels (23–25). However, research findings regarding the relationship between various circadian clock genes, psychological disorders, and sleep disorders have been inconsistent; therefore, the relationship between clock genes, psychological disorders, and sleep disorders needs to be further explored.

Mental work is a form of labor that involves external information processing, treatment, and integration through the central nervous system, as well as the transformation and output of internal information. The challenges faced by mental workers are gradually increasing due to the rapid development of science and technology. Mental workers, characterized by high cognitive load and work pressure, face a higher risk of anxiety, depression, and sleep disorders (26). Recently, more studies have been conducted on the effects of anxiety, depression, and circadian clock genes on sleep disorders, while fewer studies have been conducted on the effects of anxiety, depression, and the interaction of circadian clock genes on sleep disorders in mental workers. CLOCK and PER2 are core circadian clock genes directly involved in the regulation of the sleep–wake cycle, while RORA has been linked to circadian rhythm disruption and sleep homeostasis via its role in nuclear receptor signaling. Previous studies have primarily focused on CLOCK and PER2 in sleep disorders, but RORA’s role remains underexplored, particularly in mental worker populations. Therefore, this study investigated the current status of anxiety, depression, and sleep disorders in the mental worker population, analyzing the effects of anxiety, depression, and CLOCK, PER2, and RORA gene polymorphisms, as well as their interactions on sleep disorders to provide scientific references for the reduction of the risk of sleep disorders in the mental worker population. This study adheres to the STREGA (STrengthening the Reporting of Genetic Association studies) guidelines for transparent reporting of genetic associations. It is important to acknowledge that genetic studies may face reproducibility challenges due to population heterogeneity and multiple testing, which are addressed here via rigorous quality control and statistical corrections.

## 2 Measures

### 2.1 Subjects

In this study, questionnaires were administered, and blood samples were collected from June 2022 to February 2023 using a whole-cluster sampling method by sampling selected teachers, civil servants, medical staff members, and administrators at the Health Management Center of Xinjiang Medical University. In this study, questionnaires were distributed to a total of 2,100 mental workers. Excluding those whose questionnaires were of substandard quality, a total of 1994 questionnaires were returned, with a recovery rate of 94.95%. The inclusion criteria were (1) age ≥18 years; (2) working experience ≥1 year; (3) willingness to cooperate in the collection of blood samples after being informed of the purpose and significance of the study by the investigator. The exclusion criteria were (1) a history of physical diseases that may cause sleep disorders, such as cardiovascular and cerebrovascular diseases, diabetes mellitus, etc.; (2) a history of psychiatric diseases that may cause sleep disorders, such as bipolar disorder, schizophrenia, etc.; (3) having been treated for sleep quality problems with medication or hospitalization in the last 3 months.

Case-control matching was performed at a 1:1 ratio based on age (±5 years), gender, and educational level to minimize confounding by demographic factors. The case flow was as follows: 556 sleep disorder cases were identified from 1994 participants, and 374 cases were randomly selected and matched to 374 normal sleep controls, resulting in a total of 748 subjects for genetic analysis. The sample size for different test efficacies in case-control studies was estimated using the PASS software package; sample size was calculated based on prior studies showing that PER2 polymorphisms are associated with sleep disorders (OR = 1.5, *α* = 0.05, *β* = 0.2), which resulted in a sample size of 368, and 374 subjects in each of the case and control groups in this study, which was in line with the requirements of the sample size. The research proposal was approved by the Ethics Committee of Xinjiang Medical University (the ethical NO.20170214–174). Prior to the start of the survey, all respondents voluntarily provided written informed consent forms.

### 2.2 Self-assessment scale for anxiety

The Self-Assessment Scale for Anxiety (SAS) was developed by Zung (27), and Dunstan and Scott (28) showed that the scale had good reliability and validity in the population. The scale includes 20 items that cover a variety of anxiety symptoms, including psychological symptoms (e.g., “I feel fearful for no reason,” “I feel like I’m falling apart”) and somatic symptoms (e.g., “my arms and legs are shaking,” “I feel my heart beating rapidly”), and it instructs the study subjects to respond according to their condition over the prior week. The scale uses a 4-level scoring system (in which items 5, 9, 13, 17, and 19 are inversely scored), and the score range of each item is 1 ~ 4 points. The total score was multiplied by 1.25 to obtain the final score, and a final score of 50 points, according to the Chinese standard ≥50, indicates the occurrence of anxiety. The Cronbach’s *α* coefficient of the scale in this study is 0.75, indicating that the SAS has good reliability.

### 2.3 Self-assessment scale for depression

The Self-Assessment Scale for Depression (SDS), developed by Zung et al. (29), was used, which was shown by Jokelainen (30) and others to have good reliability and validity. The scale consists of 20 items covering a wide range of depressive symptoms that the study participants were instructed to answer based on the prior week. A 4-level scoring system was used (with 2, 5, 6, 11, 12, 14, 16, 17, 18, and 20 being reverse-scored items), with a range of 1 to 4 points assigned for each item. The total score was multiplied by 1.25 to obtain the final score, and a final score of ≥53 points suggested the occurrence of depression according to the Chinese standard. In the present study, the Cronbach’s *α* coefficient of this scale was 0.86, indicating good reliability.

### 2.4 Pittsburgh sleep quality index

The Chinese version of the Pittsburgh Sleep Quality Index (PSQI) questionnaire, developed by Buysse et al. (31), was used. This questionnaire includes the seven dimensions of subjective sleep quality, time to sleep, sleep duration, sleep efficiency, sleep disorders, hypnotic drugs, and daytime dysfunction, totaling 19 items. The seven dimensions were scored using a 4-level scoring system, with each item having a score ranging from 0 ~ 3 points. Referring to the national standard, a cumulative score greater than 7 was understood as indicating sleep disorders (32), and the Cronbach’s alpha coefficient of this scale was 0.82 in this study.

### 2.5 Determination of gene polymorphisms

Based on the relevant literature from China and abroad and the HapMap database^1^, SNP genotype information of the Chinese genome (CHB) and Northwest European ancestry (CEU, for Uyghur participation) was used in this study. The Haploview software package^2^ was used to set the corresponding running parameters. Minimum allele frequency (MAF) > 0.1 and the chain imbalance parameter, *r*^2^ > 0.8 were used to screen genetic locations. The SNP loci of the CLOCK gene (rs11932595 and rs10462028), PER2 gene (rs934945 and rs2304572), and RORA gene (rs10851687 and rs3743266) were screened and analyzed in this study. Information regarding the primers used is listed in Table 1.

**Table 1:** Primer sequences.

Gene	Genetic locus	Primer direction	Sequence 5′ → 3′
CLOCK	rs10462028	Forward	CAAAGGAGAGCACAGCACTGTCA
		Reverse	ACCGTGCTTTGGACTTGCAGTT
	rs11932595	Forward	ATCTCATCCATCTTGAGTGCATTGG
		Reverse	CTGAAGAGAGTTTAGCACAAGGAACC
PER2	rs934945	Forward	GGCAGGGGTTACGTCTGCTCTT
		Reverse	GGAAGATATTCCTTCTCTGGGACTCA
	rs2304672	Forward	GTCCACTGGAGCCACTGCTCAT
		Reverse	GCGTGACAGCATCCCTCTGTTT
RORA	rs10851687	Forward	GCAGACATTTGCACCTCCTGAC
		Reverse	CAGCCCTACCTGCATCCAGAAAT
	rs3743266	Forward	GGAAGCTTAGGATGCTAAATCGTGCT
		Reverse	CATGATTATTGGCTTCCATGCACT

### 2.6 Quality control

#### 2.6.1 Quality control of the epidemiological investigation

Strict quality control was used in both epidemiological field investigations and experimental processes. Before the questionnaires were administered, the investigators were trained on the content of the questionnaire and the method of questionnaire completion. The questionnaire numbers were matched with the same blood sample numbers. During the investigation, the investigator urged each respondent to complete the questionnaire correctly and completely, and unqualified questionnaires were re-filled or discarded.

#### 2.6.2 Laboratory quality control

DNA extraction and PCR were carried out in strict accordance with the experimental procedures. After completion of the daily experiments, the operating table and experimental supplies were sterilized.

### 2.7 Statistical analysis

In this study, Epi Data 3.1 software was used for the database creation, double entry of valid questionnaires, and logical checking. IBM SPSS STATISTICS 25.0 was used for data analysis. The normality test was performed for the measurement data, and data obeying normal distribution were described by $\bar{x}$ ± s. Further, two independent samples t-tests were used for the comparison of the means of the two groups, and an ANOVA was used for the comparison of the means of multiple groups. If there was a difference in the overall total, the SNK-q test was used for the comparison of the two. Count data were described by the rate and constitutive ratio, and the comparison of the rate was performed by the *χ*^2^ test. Associations between anxiety, depression, and sleep disorders were analyzed by partial correlation analysis and a logistic regression model was used to analyze the factors influencing sleep disorders. GMDR software was used to fit the gene–environment interaction model (test level *α* = 0.05). GMDR was chosen for interaction modeling because it effectively captures high-order gene–environment interactions, which is challenging with traditional logistic regression that primarily handles low-order or additive effects. For genetic association analyses, multiple testing corrections were performed using the Bonferroni method. The significance threshold was adjusted to *p* < 0.008 (0.05/6 SNPs) to account for the six genetic loci tested. GMDR has limitations, including potential sensitivity to sample size and difficulty in interpreting high-order interactions.

## 3 Results

### 3.1 Demographic characteristics of mental workers

The demographic characteristics of the participants are shown in Table 2.

**Table 2:** Demographic characteristics of mental workers.

Variables	Group	Number (*n*)	Ratio (%)
Sex	Male	853	42.78
	Female	1,141	57.22
Age group, years	≤30	631	31.64
	30~	1,056	52.96
	>45	307	15.40
Working years	≤10	1,047	52.51
	>10	947	47.49
Marital status	Single	565	28.34
	Married	1,384	69.41
	Divorce or widowhood	45	2.26
Shift	Fixed day shift	1,454	72.92
	Shift	540	27.08
Educational level	College degree and below	402	20.16
	Undergraduate	1,223	61.33
	Master’s degree or above	369	18.51
Professional titles	Primary	1,165	58.43
	Secondary	595	29.84
	Senior	234	11.74
Profession	Teacher	548	27.48
	Medical personnel	374	18.76
	Public servant	569	28.54
	Administrative staff	503	25.23
Monthly income	<5,000	451	22.62
	5,000 ~ 8,000	912	45.74
	>8,000	631	31.64

### 3.2 Occurrence of sleep disorders according to different population characteristics

Sleep disorders occurred in 556 (27.88%) of mental workers in this study (*p* < 0.05). The detection rate of sleep disorders varied widely with statistical significance (p < 0.05) according to gender, age, shift work, education, title, occupation, and monthly income (see Table 3).

**Table 3:** Occurrence of sleep disorders according to different population characteristics.

Variables	Group	Number	Sleep disorders (n)	Incidence rate (%)	*χ* ^2^	*p*
Sex	Male	853	181	21.22	32.925	<0.001
	Female	1,141	375	32.87		
Age group, years	≤30	631	157	24.88	27.087	<0.001
	30~	1,056	276	26.14		
	>45	307	123	40.07		
Working years	≤10	1,047	275	26.27	2.871	0.090
	>10	947	281	29.67		
Marital status	Single	565	141	24.96	7.375	0.025
	Married	1,384	396	28.61		
	Divorce or widowhood	45	19	42.22		
Shift	Fixed day shift	1,454	348	23.93	41.652	<0.001
	Shift	540	208	38.52		
Educational level	College degree and below	402	148	36.82	24.230	<0.001
	Undergraduate	1,223	329	26.90		
	Master’s degree or above	369	79	21.41		
Professional titles	Primary	1,165	357	30.64	10.853	0.004
	Secondary	595	140	23.53		
	Senior	234	59	25.21		
Profession	Teacher	548	138	25.18	22.311	<0.001
	Medical personnel	374	141	37.70		
	Public servant	569	144	25.31		
	Administrative staff	503	133	26.44		
Monthly income	<5,000	451	131	29.05	10.698	0.005
	5,000 ~ 8,000	912	279	30.59		
	>8,000	631	146	23.14		

### 3.3 The occurrence of sleep disorders in mental workers with different levels of anxiety

The results of the study showed that there was a statistically significant difference in the occurrence of sleep disorders between groups with different levels of anxiety (*χ*^2^ = 643.499, *p* < 0.001), suggesting that the occurrence of sleep disorders is associated with anxiety (see Table 4).

**Table 4:** Occurrence of sleep disorders in mental workers with different levels of anxiety.

Anxiety	*N*	Sleep disorders		*χ* ^2^	*P*
		*n*	%		
No	1,374	148	10.77	643.499	<0.001
Yes	620	408	65.81		

### 3.4 The occurrence of sleep disorders in mental workers with different level of depression

The results of the study showed that there was a significant difference in the occurrence of sleep disorders between groups with different levels of depression (*χ*^2^ = 810.588, *p* < 0.001), suggesting that the occurrence of sleep disorders is related to depression (see Table 5).

**Table 5:** Occurrence of sleep disorders in mental workers with different levels of depression.

Depression	*N*	Sleep disorders		*χ* ^2^	*P*
		*n*	%		
No	1,477	162	10.97	810.588	<0.001
Yes	517	394	76.21		

### 3.5 Partial correlation analysis of anxiety, depression, and sleep disorders in mental workers

Anxiety scores were positively correlated with the scores of all dimensions of sleep disorders (*p* < 0.05), and depression scores were positively correlated with the scores of all dimensions of sleep disorders (*p* < 0.05), suggesting that there is a positive correlation between the degree of anxiety and depression and sleep disorders and that anxiety, depression, and sleep affect each other (see Table 6).

**Table 6:** Partial correlation analysis of anxiety, depression, and sleep disorders.

Variables	PSQI total score	Subjective sleep quality	Time of fall asleep	Sleeping time	Sleep efficiency	Sleep disorders	Hypnotic drugs	Daytime dysfunction
Anxiety score	0.626**	0.465**	0.437**	0.281**	0.210**	0.512**	0.295**	0.517**
Depression score	0.661**	0.481**	0.454**	0.309**	0.271**	0.494**	0.320**	0.534**

*P < 0.05, **P < 0.001.

### 3.6 Genetic susceptibility to sleep disorders in mental workers

#### 3.6.1 Hardy–Weinberg genetic balance test

The results of the Hardy–Weinberg genetic balance test show that the actual values of the genotypes of the rs10462028 and rs11932595 of the CLOCK gene, the rs934945 and rs2304572 of the PER2 gene, and the rs10851687 and rs3743266 of the RORA gene are in good agreement with the expected values, and none of the differences were statistically significant (*p* > 0.05). It is suggested that the mental workers in the case and control groups have biological clock gene frequencies that are in accordance with the law of genetic equilibrium and that are in a state of genetic equilibrium (see Table 7).

**Table 7:** Hard–Weinberg genetic balance test.

Gene	Genetic locus	Genotype	Control group		*χ* ^2^	*P*	Case group		*χ* ^2^	*P*
			Actual value	Expected value			Actual value	Expected value		
CLOCK	rs10462028	GG	294	292.94	0.288	0.591	250	252.65	1.684	0.194
		GA	74	76.11			116	108.71		
		AA	6	4.94			8	11.65		
	rs11932595	AA	305	303.66	0.604	0.437	260	255.30	2.869	0.090
		GA	64	66.68			98	107.41		
		GG	5	3.66			16	11.30		
PER2	rs934945	CC	190	196.37	2.721	0.099	155	159.84	1.223	0.269
		CT	162	149.27			179	169.32		
		TT	22	28.37			40	44.84		
	rs2304572	GG	310	309.09	0.324	0.569	290	290.29	0.021	0.884
		GC	60	61.82			79	78.41		
		CC	4	3.09			5	5.29		
RORA	rs10851687	AA	266	261.95	2.355	0.125	262	260.28	0.415	0.520
		AT	94	102.10			100	103.44		
		TT	14	9.95			12	10.28		
	rs3743266	CC	7	9.95	1.248	0.264	9	12.55	1.502	0.220
		CT	108	102.10			119	111.91		
		TT	259	261.95			246	249.55		

#### 3.6.2 Correlation study of CLOCK, PER2, and RORA genes with sleep disorders

The distribution frequencies of each genotype and allele of the rs10462028 and rs11932595 of the CLOCK gene and the rs934945 of the PER2 gene were different in the distributions of case and control groups (*p* < 0.05). The rs2304572 of the PER2 gene, the rs10851687 of the RORA gene, and the rs3743266 showed no difference in the distribution frequency of each genotype and allele (*p* > 0.05) (see Table 8).

**Table 8:** Correlation study of CLOCK, PER2, and RORA genes with sleep disorders.

Gene	Genetic locus	Genotype	Control group	Case group	*χ* ^2^	*p*
CLOCK	rs10462028	GG	294	250	13.129	0.001
		GA	74	116		
		AA	6	8		
		G	622	616	11.362	0.001
		A	86	132		
	rs11932595	AA	305	260	16.482	<0.001
		AG	64	98		
		GG	5	16		
		A	674	618	17.800	<0.001
		G	74	130		
PER2	rs934945	CC	190	155	9.624	0.008
		CT	162	179		
		TT	22	40		
		C	542	489	8.765	0.003
		T	206	259		
	rs2304572	GG	310	290	3.375	0.185
		GC	60	79		
		CC	4	5		
		G	680	659	3.138	0.076
		C	68	89		
RORA	rs10851687	AA	266	262	0.370	0.831
		AT	94	100		
		TT	14	12		
		A	626	624	0.019	0.889
		T	122	124		
	rs3743266	CC	7	9	1.118	0.572
		CT	108	119		
		TT	259	246		
		C	122	137	1.051	0.305
		T	626	611		

### 3.7 Gene–environment interactions for sleep disorders

#### 3.7.1 GMDR model of gene–environment interaction

The GMDR software was used to build the interaction model for analyzing the effect of the one-way statistically significant interaction between rs10462028, rs11932595, rs934945, anxiety, and depression on sleep disorders. As shown in Table 9, the fitted interaction model between rs934945, anxiety, and depression was optimal with *p* = 0.0010, a cross-validation consistency of 10/10, and a test sample accuracy of 0.8535.

**Table 9:** GMDR model of sleep disorders with environment–gene interactions.

Model	Testing balance accuracy	Sign Test (P)	CV consistency
Anxiety	0.8533	0.0010	9/10
Anxiety*Depression	0.8520	0.0010	9/10
rs934945*Anxiety*Depression	0.8535	0.0010	10/10

#### 3.7.2 Gene–environment interaction on sleep disorders — logistic regression analysis

Logistic regression model fit was evaluated via Hosmer-Lemeshow test (*χ*^2^ = 5.32, *p* = 0.72), indicating good agreement between observed and predicted values. The results of the gene–environment interaction regression analysis showed that compared with the CC genotype at rs934945, the CT/TT genotype (OR = 1.442, 95% CI: 1.077 ~ 1.931) increased the risk of sleep disorders occurring in mental workers, and compared with those who had no anxiety and those who had no depression, those who were anxious (OR = 4.630, 95% CI: 1.912 ~ 11.211) or depressed (OR = 10.160, 95% CI: 7.245 ~ 14.248) had a higher risk of sleep disorders. The interaction of rs934945, anxiety, and depression (OR = 10.461, 95% CI: 3.695 ~ 29.621) increased the risk of sleep disorders in mental workers (see Table 10).

**Table 10:** Logistic regression analysis of gene–gene and gene–environment interactions on sleep disorders.

Variables	Compare groups	Reference group	*β*	wald	*P*	OR (95%CI)
rs10462028	GA/AA	GG	0.307	1.509	0.219	1.359(0.833 ~ 2.217)
rs11932595	AG/GG	AA	0.411	2.505	0.113	1.508(0.907 ~ 2.510)
rs934945	CT/TT	CC	0.366	6.025	0.014	**1.442(1.077 ~ 1.931)**
Anxiety	Yes	No	1.533	11.537	0.001	**4.630(1.912 ~ 11.211)**
Depression	Yes	No	2.318	122.817	<0.001	**10.160(7.245 ~ 14.248)**
rs10462028*rs11932595*rs934945			1.305	17.872	<0.001	**2.816(1.742 ~ 4.550)**
rs934945*Anxiety*Depression			2.348	19.545	<0.001	**10.461(3.695 ~ 29.621)**

Bold indicates *P* < 0.05.

## 4 Discussion

The detection rate of sleep disorders was 27.88% in this study including 1944 mental workers. Niu et al.’s (33) study on the relationship between burnout and sleep quality of teachers in undergraduate colleges and universities in Inner Mongolia found that the positive rate of sleep disorders was 12.4%, and Lv et al.’s (34) study on the sleep quality of workers in the railroad vehicle system showed that the detection rate of sleep disorders was 20.47%. When compared with the above results of the studies on the sleep disorders of these occupational groups, the sleep disorders of mental workers in Xinjiang were more serious. However, with the rapid development of the economy, mental workers, as the main force of productivity, face an increasingly fast-paced life and work environment, and various kinds of overtime work, shift work, and increasing work pressure have seriously affected their sleep time and quality (35, 36).

The number of female mental workers in this study who developed sleep disorders was 357 (32.78%), and the detection rate of sleep disorders among them was higher than among male workers, a result that is consistent with the findings of Fu et al. (37). This may be due to the fact that compared with men, working women need to balance family and work, and under this dual pressure, they are prone to experiencing work–family conflicts, which may further lead to psychological disorders that can, in turn, have an impact on sleep quality (38). In addition, differences in the functioning of the endocrine system between men and women also influence gender differences in sleep quality outcomes. Estrogen modulates GABA receptor sensitivity to maintain sleep. Pre-menstrual and menopausal estrogen declines disrupt sleep continuity (39). Females exhibit stronger stress-induced cortisol responses, which interfere with circadian gene (e.g., PER2) expression, creating a “hormone-psychology-gene” feedback loop. The present study also found that the prevalence of sleep disorders and the related scores on all dimensions were higher among mental workers working shifts than among those working regular day shifts. A cross-sectional study on the relationship between shift work and sleep disorders among Chinese workers by Zhang et al. (40) found that the prevalence of sleep disorders was significantly higher among workers engaged in shift work than among workers working regular day shifts. The reason for this may be that shift work causes occupational groups to reverse day and night, as well as work under bright lights at night, resulting in an imbalance of their circadian rhythms that affects their wake–sleep cycle and leads to sleep disorders (41). In this study, we found that the detection rate of poor sleep quality among divorced or widowed mental workers was higher than that of unmarried and married people. The reason may be that they not only lack social support but also suffer from emotional shock, leading to a higher prevalence of sleep disorders. Regarding the effects of different marital statuses on sleep quality, the findings of different studies vary. Some scholars have found that unmarried workers have a higher prevalence of sleep disorders due to their younger age, lack of work experience, and lower social support (42); other studies have found a higher detection rate of sleep disorders among married workers (43).

In this study, we investigated the occurrence of different anxiety conditions and sleep disorders in 1994 mental workers and found that the detection rate of sleep disorders in the anxiety group was much higher than that in mental workers without anxiety, and studies, such as that by Guo et al. (44), have also shown that the presence of negative emotions, such as anxiety, increases the incidence of sleep disorders. Further analysis of the partial correlation between the anxiety scores and sleep quality scores of mental workers showed a positive correlation between anxiety scores and scores on all dimensions of the sleep quality scores, as well as Korkmaz et al.’s (45) study on healthcare workers. It is suggested that the more severe the level of anxiety, the higher the likelihood of sleep disorders, and that the level of sleep quality can be improved by reducing the level of anxiety and other adverse emotions in mental workers, thereby improving their sleep quality. The present study investigated the occurrence of different depressive conditions and sleep disorders in 1994 mental workers and found that the incidence of sleep disorders in depressed patients was 76.21%, which was much higher than the incidence of sleep disorders in the non-depressed population (10.97%). The results of the current study also showed a positive correlation between the depression scores and sleep disorder scores of mental workers, which is consistent with the findings of other scholars (46). Given these results, alleviating depression among mental workers through reasonable ways may reduce the incidence of their sleep disorders to a greater extent. It is important to note that the cross-sectional design of this study limits our ability to establish causal relationships. The observed associations between genetic polymorphisms, anxiety, depression, and sleep disorders reflect correlations rather than direct causation. Longitudinal studies are needed to confirm the temporal sequence of these associations.

In recent years, as scholars have gradually delved deeper into the study of sleep, it has been found that the optimal quality of sleep required for the organism to maintain physiological homeostasis is affected by demographic characteristics, in addition to genetic and environmental factors. Studies have shown that the percentage of sleep quality determined by genetic factors ranges from 31 to 55%, which suggests that genetic factors have a significant influence on sleep quality (47) and that the occurrence of sleep disorders may be caused by gene–environment interactions (48). In this study, we found that anxiety, depression, and polymorphism at the rs934945 of the PER2 gene were risk factors for the development of sleep disorders. The PER2 gene is a core component of the circadian clock, encoding a key protein that regulates the sleep–wake cycle. The rs934945 polymorphism in PER2 may affect the transcriptional regulation or protein stability of PER2, thereby disrupting circadian rhythmicity. Previous studies have shown that variants in PER2 are associated with delayed sleep phase disorder and altered sleep homeostasis (49). The rs934945 SNP might influence the interaction between PER2 and other clock genes (e.g., CLOCK, BMAL1), leading to dysregulation of the circadian network and increased susceptibility to sleep disorders when combined with psychological stressors like anxiety and depression. The results of this study also suggest that changes in PER2 gene expression can affect sleep. Gene–environment interactions arise from different responses of individuals to environmental stimuli, depending on their genotype, or from different genetic effects between individuals due to differences in their living and working environments. Further exploration of the possible gene–environment interactions on the effects of sleep disorders and the identification of gene–environment interactions could potentially ameliorate the risk of the development of sleep disorders and help unravel the underlying biological pathways.

The results of the gene–environment modeling showed that the model constructed by rs934945 with anxiety and depression was optimal and that there may be an interaction between rs934945, anxiety, and depression on the generation of sleep disorders. Loci rs10462028, rs11932595, and rs934945, as well as anxiety and depression, were included as dependent variables in the logistic regression equations, and the results showed that the polymorphism of the rs934945 of the PER2 gene, anxiety, and depression was associated with a higher prevalence of sleep disorders in mental workers. The polymorphism of rs10462028 and rs11932595 of CLOCK gene had no effect on sleep disorders, which is different from the findings of Vanderlind et al. (50). Therefore, the effects of the rs10462028 and rs11932595 of the CL OCK gene on sleep disorders need to be demonstrated by further research. The interaction of rs934945, anxiety, and depression in the logistic regression results showed that the interaction of rs934945, anxiety, and depression significantly increased the prevalence of sleep disorders (OR = 10.461, 95% CI: 3.695–29.621), and the results were the same as those of the model constructed by GMDR software, which may be attributed to the fact that individuals with susceptibility genes are more likely to be adversely affected by negative environmental factors (51). Recent research has elaborated on the neurobiological links between sleep disturbances and mood disorders, highlighting that circadian gene dysregulation (e.g., PER2 variants) may disrupt serotonin and cortisol pathways, which are also implicated in anxiety and depression (52). This aligns with our finding that rs934945 interacts with anxiety/depression, potentially via shared neuroendocrine pathways. Realizing that the existence of interactions between biological clock genes and anxiety and depression can lead to an increased risk of sleep disorders in mental workers, suggests that, as a potential biological pathway, gene–environment interactions should be paid attention to in terms of their impact on sleep disorders.

These findings have practical implications for targeted interventions. For mental workers carrying the PER2 rs934945 CT/TT genotype, prioritizing psychological support (e.g., cognitive-behavioral therapy for anxiety and depression) may mitigate sleep disorder risk by addressing the gene–environment interaction. Workplace policies could also include circadian-aligned shift schedules (avoiding frequent night shifts) for this subgroup, as their genetic susceptibility may amplify circadian disruption from irregular work hours. Additionally, regular screening combining psychological assessments (SAS/SDS) and genetic testing could identify high-risk individuals for early intervention.

This study offers three key advantages. First, it is among the first to systematically investigate the interactive effects of anxiety, depression, and circadian clock gene polymorphisms (CLOCK, PER2, RORA) on sleep disorders in mental workers. Unlike prior studies that analyzed psychological or genetic factors in isolation, we confirmed via GMDR modeling and logistic regression that the interaction between rs934945 (PER2) and anxiety/depression significantly elevates sleep disorder risk (OR = 10.461, 95% CI: 3.695–29.621), highlighting a “gene-psychology” synergistic effect. Second, the large sample size (n = 1994) and multi-dimensional assessments (SAS, SDS, PSQI scales plus genotyping) enhance result reliability compared to single-scale or small-sample studies. The reliability of our assessments is supported by recent validation studies confirming that SAS, SDS, and PSQI are robust tools for evaluating mental health and sleep quality in occupational populations, with consistent psychometric properties across diverse cultural contexts (53). Third, while RORA gene variants showed no independent association, this study introduces a novel focus on RORA’s role in circadian-sleep networks, paving the way for future research.

This study has several limitations. First, its cross-sectional design precludes causal inference, necessitating longitudinal cohort studies to validate the temporal sequence of “gene-psychology-sleep” associations. Second, the sample primarily comprises mental workers from select Xinjiang enterprises, with insufficient ethnic minority representation, limiting generalizability to broader populations. Third, unadjusted lifestyle factors (e.g., physical activity, caffeine intake, medication use) may confound the observed relationships. Fourth, self-reported questionnaires are susceptible to recall bias. Future research should include multi-ethnic samples, objective sleep monitoring (e.g., polysomnography), and longitudinal designs to clarify mechanisms.

Again, the cross-sectional design precludes conclusions about causality. For example, we cannot determine whether anxiety/depression precedes sleep disorders or vice versa, nor can we confirm that rs934945 directly modulates this relationship-longitudinal cohort studies tracking genetic, psychological, and sleep parameters over time are needed to clarify temporal sequences.

## 5 Conclusion

In summary, this study found that the occurrence of anxiety, depression, and sleep disorders is serious among mental workers in Xinjiang and that the polymorphisms of anxiety, depression, and PER2 genes and their interactions have an impact on the occurrence of sleep disorders. The situation of sleep disorders among mental workers in Xinjiang is very serious, so the relevant departments and workplaces should develop corresponding policies and measures to rationally arrange work tasks and shift systems, while at the same time carrying out targeted psychological counseling and lectures to alleviate mental workers’ anxiety, depression, and other negative emotions. Simultaneously, their self-confidence and ability to positively cope with problems should be cultivated, so as to reduce the effects of anxiety and depression on sleep disorders and further promote the development of occupational groups to reduce sleep disorders and improve physical and mental health in the working population. The scales used in this study are internationally used scales with good reliability and validity, thus minimizing bias.

## Data Availability

The raw data supporting the conclusions of this article will be made available by the authors, without undue reservation.

## Footnotes

^1^ https://www.internationalgenome.org/

^2^ http://www.broad.mit.edu/mpg/haploview
